# Variants of unknown significance are common in brushite stone formers undergoing genetic testing for nephrolithiasis

**DOI:** 10.1007/s00240-025-01859-1

**Published:** 2025-09-29

**Authors:** Tomas Paneque, Kyle Berst, Meenakshi Sambharia, Melissa Swee, Mycah Kimble, M. Adela Mansilla, Margaret Freese, Christie P. Thomas, Chad R. Tracy, Ryan L. Steinberg

**Affiliations:** 1 Department of Urology, University of Iowa Health Care, 200 Hawkins Drive, 3RCP, Iowa City, IA 52242 USA; 2 Carver College of Medicine, University of Iowa, Iowa City, IA USA; 3 Department of Internal Medicine, University of Iowa Health Care, Iowa City, IA USA; 4 Iowa Institute for Human Genetics, University of Iowa, Iowa City, IA USA

**Keywords:** Genetics, Brushite, Kidney stones, Hypophosphatemic rickets

## Abstract

Up to 40% of all kidney stone disease may have an underlying genetic contribution. Brushite stone formers are known to have high rates of stone recurrence and urine chemistry abnormalities, suggesting a possible intrinsic kidney abnormality which may contribute to brushite stone formation. In this study, we sought to determine the burden of genetic variants amongst brushite stone formers. An IRB approved prospective study of brushite stone formers was conducted. Patients with any brushite on stone analysis were eligible for enrollment. Genetic testing was done on peripheral blood derived DNA and assessed using a clinically available nephrolithiasis gene panel. Primary analysis assessed rates of genetic variants amongst brushite stone formers. Secondary analysis assessed for distinct patterns in urine chemistries in different genotypes. 15 patients (8 males) with a median age of 32 years (range 22–69) underwent genetic testing. 12 patients (80%) were reported to have a genetic variant, with 6 (40%) having multiple variants. Two patients had a heterozygous pathogenic or likely pathogenic variant in a gene that causes autosomal recessive disease. 9 (60%) patients had a variant of unknown significance. 10 (67%) patients had genetic variants associated with hypophosphatemic rickets. Genetic variants of unknown significance are common in brushite stone formers. Further work is needed to assess the significance of these variants and their role in the lithogenic process.

**Supplementary Information:**

The online version contains supplementary material available at 10.1007/s00240-025-01859-1.

## Introduction

The pathophysiology of kidney stone disease is complex, multifactorial and remains incompletely understood. A family history of stone disease has been shown to be present in 35–65% of people with kidney stones while only 5–20% of non-stone formers report a family history [1–3]. Based on this data, a genetic underpinning to stone disease has long been suspected. While a monogenic disease was classically thought to account for ~ 2% of all stone disease, in one study with patients enrolled from three, high volume stone clinics, single-gene cause of nephrolithiasis was found in ~ 15% of patients (11% adult, 17–29% in pediatrics) [4]. A more recent review has suggested that up to 40% of all kidney stone disease may have an underlying genetic contribution, though the authors did not differentiate between various stone types [5]. 

Brushite (calcium monohydrogen phosphate) stones account for approximately 1.3% of all urinary stones [6]. Though relatively uncommon, brushite stone formers can be notoriously difficult to treat. Patients often have multiple stones and this stone type has shown resistance to shock wave lithotripsy, limiting surgical options [7]. Further, brushite stone formers have higher rates of recurrence [8]; this is thought to be related to the high rates of predisposing urine chemistry abnormalities, including hypercalciuria, hyperphosphaturia and a relatively alkaline urine [9, 10]. 

To date, there are no studies assessing the rate at which relevant genetic variants occur amongst brushite stone formers. We aimed to perform a pilot evaluation to assess the rate of genetic abnormalities amongst brushite stone formers. In this pilot study, we hypothesized that >50% of brushite stone formers will have genetic abnormalities including pathogenic and likely pathogenic variants and variants of uncertain significance (VUS).

## Materials and methods

### Study design

An IRB approved, prospective study of brushite stone formers was undertaken from January 2024 to July 2024. Patients with prior stone removal surgery or spontaneous stone passage with confirmed brushite stone composition on stone analysis (regardless of the percentage) were asked to participate in the study. Stone analysis was performed by an outside commercial lab via quantitative reflectance Fourier transform infrared spectroscopy. Eligible patients were approached during clinic visits to discuss study enrollment. Those interested were consented and underwent venipuncture phlebotomy. 6 mL of whole blood was collected in an EDTA containing tube. Specimens were then stored in a refrigerator (5 °C) until transport to the Iowa Institute of Human Genomics (IIHG). Samples underwent next generation sequencing (NGS) using the KidneySeq™ platform (IIHG, Iowa City, IA), a clinically validated comprehensive renal gene panel which over 330 genes that cover about 120 distinct renal diseases [11]. For this study, bioinformatic processing was limited to the nephrolithiasis/nephrocalcinosis subpanel, which consists of 37 genes contributing to 21 monogenic diseases presenting with nephrolithiasis and/or nephrocalcinosis (Supplemental Table 1). Bioinformatics analysis and variant filtering was performed on dedicated computing.

resources maintained by the Iowa Institute of Human Genetics using a standardized workflow for sequence analysis and variant calling. Library quality was based on the total number of reads per sample and coverage at 30X or greater, excluding low-quality variants (depth < 10 or quality by depth (QD) < 5), common variants with a minor allele frequency (MAF) > 1% in any population (except for known risk alleles).

Supplemental Table 1. List of genes tested in the KidneySeq™ platform (IIHG, Iowa City, IA) nephrolithiasis/nephrocalcinosis subpanel.

Patient level data was extracted from the electronic medical records of study participants including demographics, comorbidities, blood/urine studies and procedural data. Data was housed in a secured electronic RedCap database (NIH CTSA UM1TR004403).

### Classification of genetic variants

Variants in genes were categorized based on gene function (enzyme catalyst, ion/mineral transport, other), zygosity, minor allele frequency and pathogenicity prediction score. Using the American College of Medical Genetics and Genomics (ACMG) criteria and after clinical interpretation by an expert panel, including physicians, genetic counselors and bioinformaticians, variants were classified into one of the following categories: pathogenic, likely pathogenic, variant of uncertain significance (VUS)), likely benign, or benign [12]. Briefly, ‘pathogenic’ refers to a variant that is highly likely (>99% probability) and ‘likely pathogenic’ refers to a variant that is likely (90–99%) to be disease causing. Likely benign and benign are at the other end of the spectrum and respectively have a 1–10% and < 10% probability of being disease causing. VUS covers all the variants in between (>10% <90%) that cannot be further interpreted because of a lack of evidence that permit upgrading or downgrading towards pathogenicity or benignity. A point-based system supporting the categorization as above has been previously reported [13].

### Analysis

Descriptive statistics were generated based on results of gene testing and demographic data. Initial 24-hour urine chemistries, prior to initiation of any medical therapy, were compared using the t-test (*p* < 0.05).

## Results

### Cohort

A total of 15 patients (8 males) with a median age of 32 years (range 22–69 years of age) underwent genetic testing. 14 (93%) patients had a history of recurrent stone disease with 11 (73%) of these patients requiring prior surgery. 4 (27%) patients reported a family history of kidney stone disease. Table 1 specifies baseline demographics of this cohort.

**Table 1:** Patient demographics of brushite stone formers undergoing genetic testing

	Number	Percentage
Gender		
Male	8	53%
Female	7	47%
Age		
18 or below	0	0%
≥ 18 and ≤ 25	3	20%
≥ 26 and ≤ 35	8	53%
≥ 36 and ≤ 45	2	13%
≥ 46 and ≤ 55	1	7%
≥ 56 and ≤ 65	0	0%
66 or above	1	7%
BMI		
18.5 or below	0	0%
>18.5 and ≤ 24.9	4	27%
>25 and ≤ 29.9	5	33%
>30 and ≤ 39.9	4	27%
40 or above	2	13%
Family history of stones	4	27%
Number with history prior stone events	14	93%
Median number of prior stone events (IQR)	10 (2–10)	-
History of prior stone surgery	11	73%
Median number of prior stone surgeries (IQR)	3 (1–4)	-
History of diabetes mellitus type 2	2	13%
History of gout	0	0%
History of Crohn’s	0	0%
History of ulcerative colitis	1	7%
History of chronic diarrhea	1	7%
History of celiac disease	0	0%
History of pancreatitis	0	0%
History of sarcoidosis	0	0%
History of distal renal tubular acidosis	0	0%
History of hyperparathyroidism	0	0%
History of recurrent urinary tract infections	4	27%
History of medullary sponge kidney	1	7%
History of bariatric surgery	0	0%

### Genetics

Twelve (80%) brushite stone formers harbored heterozygous genetic variants, of which 6 (40%) patients had multiple variants. No patients had pathogenic or likely pathogenic variants sufficient to cause monogenic disease. However, 2 patients were heterozygous carriers of a single pathogenic or likely pathogenic variant in genes associated with diseases of autosomal recessive inheritance. 10 (67%) patients harbored genetic variants in genes associated with dominant and recessive hypophosphatemic rickets; 6 (40%) had specific variants in *SLC34A3*, which is causal of hereditary hypophosphatemic rickets with hypercalciuria (HHRH). A total of 10 (66%) patients harbored VUSs in several genes. The identified variants are shown in Table 2.

**Table 2:** List of brushite stone patients with associated genetic variants identified on testing for nephrolithiasis/nephrocalcinosis disorders

Patient	Gene	Variant coordinates (hg19)	HGSV Nomenclature	Gene Classification	Classification	ACMG Criteria[12]	Scale Point System[13]	Associated Diseases
1	*CYP24A1*	20:52789454:A > G	NM_000782.5:c.443T > C, p.Leu148Pro	Enzyme catalyst	Pathogenic ^	PM3, PP1, PS3, PM2, PP3	9 (Likely Pathogenic)	Infantile hypercalcemia 1 (AR)
	*SLC12A1*	15:48548063:T > C	NM_000338.3:c.1998T > C, p.(Asn666=)	Ion/mineral transport	VUS	PM2, BP7,	1 (VUS)	Bartter Syndrome (AR)
	*SLC4A1*	17:42334820:C > G	NM_000342.4:c.1524G > C, p.(Glu508Asp)	Ion/mineral transport	VUS	PM2	2 (VUS)	Distal RTA (AD & AR)
2	*FGF23*	12:4488611 T > C	NM_020638.3:c.138 A > G, p.(Thr46=)	Ion/mineral transport	VUS	PM2, BP7	1 (VUS)	Hypophosphatemic Rickets (AD)
3	No variant							
4	*ADCY10*	1:167825477 T > C	NM_018417.6:c.2097 A > G, p.(Ala699=)	Enzyme catalyst	Benign	BS1, BS2, BP7	−9 (Benign)	Absorptive hypercalciuria (AD)^#^
	ENPP1	6:132203498 C > T	NM_006208.3:c.2114 C > T, p.(Thr705Met)	Enzyme catalyst	VUS	PM2	2 (VUS)	Hypophosphatemic Rickets (AR)
5	*HNF4A*	20:43048416 G > A	NM_000457.4:c.792G > A, p.(Val264=)	Enzyme catalyst	VUS	BP7	−1 (Likely Benign)	Fanconi Renotubular syndrome (AD)
6	*HNF4A*	20:42984316:T > C	Upstream untranslated region	Enzyme catalyst	Benign	PM2, BP7, BP6	2 (VUS)	Fanconi Renotubular syndrome (AD)
	*SGK3*	8:67748014 G > A	NM_001033578.3:c.542G > A, p.(Arg181Gln)	Ion/mineral transport	VUS	PM2, BP4	− 1 (Likely Benign)	Hypophospatemic Rickets (AD)*
	*SLC12A1*	15:48541795 A > G	NM_000338.3:c.1708 A > G, p.(Ile570Val)	Ion/mineral transport	VUS	PM2	2 (VUS)	Bartter Syndrome (AR)
	*SLC4A1*	17:42335485 C > T	NM_000342.4:c.1151G > A, p.(Arg384His)	Ion/mineral transport	VUS	BS1	−4 (Likely Benign)	Distal RTA (AD & AR)
7	*EHHADH*	3:184910069 T > A	NM_001966.4:c.2117 A > T, p.(Asn706Ile)	Other	VUS	PM2, BS2	−2 (Likely Benign)	Fanconi Renotubular syndrome (AD)
	*KCNJ10*	1:160011231 T > C	NM_002241.5:c.1092 A > G, p.(Gln364=)	Ion/mineral transport	Benign	BA1, BS2, BP7	−13 (Benign)	SESAME syndrome (AR)
	*SLC34A3*	9:140128107 G > A	NM_001177316.2:c.779G > A, p.(Ser260Asn)	Ion/mineral transport	Likely Benign	BS1, BS2_sup, BP4	−6 (Likely Benign)	Hypophosphatemic rickets with hypercalciuria (AR)
8	*SLC34A3*	9:140127124 C > T	NM_001177316.2:c.273 C > T, p.(Asp91=)	Ion/mineral transport	Likely benign	BS1, BS2_sup, BP7	−6 (Likely Benign)	Hypophosphatemic rickets with hypercalciuria (AR)
9	*SLC34A3*	9:140127675 C > T	NM_001177316.2:c.575 C > T, p.Ser192Leu	Ion/mineral transport	Likely pathogenic ^	PM3, PM5, PP1, PP3	6 (Likely Pathogenic)	Hypophosphatemic rickets with hypercalciuria (AR).^$ %^
	*SLC3A1*	2:44508595 G > A	NM_000341.4:c.680G > A, p.(Arg227Gln)	Ion/mineral transport	VUS	PM2, PM5, PP3	5 (VUS)	Cystinuria (AD & AR)
10	*ALPL*	1:21894692 C > T	NM_000478.6:c.744 C > T, p.(Asp248=)	Ion/mineral transport	Likely benign	PM2, BP7, BP6	0 (VUS)	Hypophosphtatasia (AD & AR)
	*SLC34A3*	9:140130522 G > A	NM_001177316.2:c.1454G > A, p.(Arg485His)	Ion/mineral transport	Likely benign	BS1, BS2_sup,	−5 (Likely Benign)	Hypophosphatemic rickets with hypercalciuria (AR)
	*SLC34A3*	9:140130653	NM_001177316.2:c.1585 A > T, p.(Ile529Phe)	Ion/mineral transport	Likely benign	BS1, BS2_sup, BP4	−6 (Likely Benign)	Hypophosphatemic rickets with hypercalciuria (AR)
11	*DMP1*	4:88583774 C > A	NM_004407.4:c.844 C > A, p.(Leu282Ile)	Ion/mineral transport	Likely Benign	BS1, BS2, BP4, BP6	−10 (Benign)	Hypophosphatemic rickets (AR)
12	*SLC34A3*	9:140128917 G > A	NM_001177316.2:c.1143G > A, p.(Ala381=)	Ion/mineral transport	VUS	PM2, BP6	0 (VUS)	Hypophosphatemic rickets with hypercalciuria (AR)
13	No variant							
14	*SLC4A1*	17:42337247 C > T	NM_000342.4:c.539G > A, p.(Arg180His)	Ion/mineral transport	Benign	BS1, BS2	−8 (Likely Benign)	Distal RTA (AD & AR)
	*ADCY10*	1:167825477 T > C	NM_018417.6:c.2097 A > G, p.(Ala699=)	Ion/mineral transport	Benign	BS1, BS2, BP6, BP7	−10 (Benign)	Absorptive hypercalciuria (AD)^#^
	*SLC34A3*	9:140128157 G > A	NM_001177316.2:c.829G > A, p.(Gly277Ser)	Ion/mineral transport	VUS	PM2, BP4	0 (VUS)	Hypophosphatemic rickets with hypercalciuria (AR)
15	No variant							

*Gene disease association not confirmed; #Gene variants increase susceptibility to disease; ^ Single pathogenic or likely pathogenic variant in an autosomal recessive gene. ^$^ This variant has been reported in patients with AR disease. ^%^ Some individuals with a single copy of this variant present with milder hypercalciuria and nephrolithiasis (PMIDs: 19820004 and 30798342)

het= heterozygous, VUS= Variant of Unknown Significance, AD= Autosomal Dominant, AR= Autosomal Recessive

### 24-Hour urine results

Of the 12 patients with genetic abnormalities, 11 went on to complete at least one 24-hour urine collection. Table 3 demonstrates the specific genetic abnormalities with associated serologic and 24-hour urine chemistry results.

**Table 3:** Serologic and 24 h urine results in patients with genetic variants on nephrolithiasis panel testing (KidneySeq)

	Genetic Results		Serum Studies							24 h Urine Studies										
Patient	Gene	Associated diseases	Sodium (mEq/L)	Potassium (mEq/L)	Bicarbonate (mEq/L)	Creatinine (mg/dL)	Calcium (mg/dL)	Magnesium (mg/dL)	Phosphate (mg/dL)	Volume (L/day)	pH	Sodium (mmol/day)	Calcium (mg/day)	Oxalate (mg/day)	Citrate (mg/day)	Ammonia (mmol/day)	Sulfate (mEq/day)	Magnesium (mg/day)	Phosphate (g/day)	Supersaturation Calcium Phosphate
1	CYP24A1	Infantile hypercalcemia (AR)	136	3.6	20	0.6	9.7	1.7	3	1.06	6.196	184	298	24	497	31	27	90	0.856	3.79
	SLC12A1	Bartter Syndrome (AR)																		
	SLC4A1	Distal RTA (AD & AR)																		
2	FGF23	Hypophosphatemic Rickets (AD)	140	4	26	1.4	9.5	2	3.7	0.76	6.726	83	54	28	378	11	23	95	0.52	1.59
4*	ADCY10	Absorptive hypercalciuria (AD)#	138	3.9	23	1.1	-	-	-	1.22	5.841	145	194	25	214	40	45	55	0.898	1.25
	ENPP1	Hypophosphatemic Rickets (AR)																		
5	HNF4A	Fanconi Renotubular syndrome (AD)	137	3.8	22	0.5	8.9	1.9	3.1	-	-	-	-	-	-	-	-	-	-	-
6	HNF4A	Fanconi Renotubular syndrome (AD)	141	3.6	28	1	9.3	-	-	2.79	6.552	337	191	47	389	60	49	142	1.196	1.05
	SGK3	Hypophospatemic Rickets (AD)+																		
	SLC12A1	Bartter Syndrome (AR)																		
	SLC4A1	Distal RTA (AD & AR)																		
7*	EHHADH	Fanconi Renotubular syndrome (AD)	140	4	30	0.7	9.5	2.2	3.7	1.78	6.703	108	231	24	543	24	32	92	0.623	2.26
	KCNJ10	SESAME syndrome (AR)																		
	SLC34A3	Hypophosphatemic rickets with hypercalciuria (AR)																		
8	SLC34A3	Hypophosphatemic rickets with hypercalciuria (AR)	135	4	23	0.9	9	1.9	3.2	3.76	6.655	166	455	41	379	24	32	178	1.147	2.27
9*	SLC34A3	Hypophosphatemic rickets with hypercalciuria (AR)	138	4.6	23	0.8	9.6	1.8	2.9	1.19	6.339	217	261	31	265	32	43	98	0.924	3.3
	SLC3A1	Cystinuria (AD & AR)																		
10*	ALPL	Hypophosphtatasia (AD & AR)	141	4	26	1	9.1	2.3	3.1	1.32	6.062	227	206	25	505	32	45	107	1.179	2.28
	SLC34A3	Hypophosphatemic rickets with hypercalciuria (AR)																		
	SLC34A3	Hypophosphatemic rickets with hypercalciuria (AR)																		
11	DMP1	Hypophosphatemic rickets (AR)	139	3.7	22	1	8.4	-	-	1.57	6.497	204	275	18	157	36	16	80	0.643	2.43
12*	SLC34A3	Hypophosphatemic rickets with hypercalciuria (AR)	139	3.5	22	1	-	-	-	1.92	6.127	175	121	23	160	32	20	84	0.954	0.79
14	SLC4A1	Distal RTA (AD & AR)	141	3.9	30	1	9.3	-	-	1.76	7.31	312	278	28	638	39	50	126	1.028	3.78

"-" demarcates missing data

*denotes patients with 2, 24 hour urine collections with reported results being the average of the two

+Gene disease association not confirmed; #Gene variants increase susceptibility to disease

Notably, 7/11 patients had urine with a pH > 6.2, 5/11 patients had hypercalciuria (> 250 mg/day), 10/11 patients had hypocitraturia (< 550 mg/day) and 6/11 patients had high normal urinary phosphate (normal = 0.6–1.2 g/day). Due to the low number of patients without genetic variants and those with non-HHRH variants, no subgroup comparisons between 24-hour urine chemistries were attempted. Five participants with *SLC34A3* variants and 3 with other variants associated with other forms of hypophosphatemic rickets completed 24-hour urine collections. Given the small number in each group, no statistical comparisons were undertaken. Though, there were notable differences between the groups with regards to urine volume, calcium and phosphate levels (Table 4).

**Table 4:** Baseline 24-Hour urine chemistry profiles of brushite stone formers with genetic variants for hypophosphatemic rickets

	SLC34A3 Variants (*n* = 5)	IQR	Other Variants (*n* = 3)	IQR
Volume (L)	2.1	(1.4–3.3)	1.43	(1.1-2.0)
Creatinine (mg)	1770	(1251–1777)	1906	(1401–1916)
Urine pH	6.55	(6.42–6.71)	6.56	(6.26–6.64)
Sodium (mmol)	216	(139–273)	206	(144–244)
Calcium (mg)	288	(219–365)	183	(118–247)
Oxalate (mg)	36	(22–44)	28	(27–52)
Citrate (mg)	491	(199–495)	570	(474–704)
Sulfate (mEq)	38	(20–45)	32	(28–42)
Ammonia (mmol)	37	(27–50)	25	(18–31)
Magnesium (mg)	129	(88–160)	123	(109–159)
Potassium (mmol)	54	(38–64)	60	(47–82)
Phosphorus (g)	1.100	(0.619–1.172)	1.391	(0.955–1.589)
Uric Acid (g)	0.770	(0.520–0.953)	0.658	(0.475–0.667)
SS CaOx	6.69	(4.43–8.43)	6.35	(5.94–8.02)
SS CaPhos	2.47	(1.66–2.67)	2.27	(1.93–2.59)
SS Uric Acid	0.59	(0.10–0.40)	0.17	(0.17–0.57)

*All values are medians

## Discussion

With improved technologies, including next-generation sequencing, the threshold to better understand genetic underpinnings of disease has continued to be lowered. This is no truer than in kidney stone disease. Large population, genome wide association studies have helped to elucidate common genetic variants and molecular pathways associated with kidney stones [14, 15]. According to one review, there are now 46 genes that cause monogenic kidney stones, with disease association or risk in another 23 genes, as well as evidence for polygenic causes [5]. The ability to bring genetic testing into the clinical realm is hampered by a lack of understanding of which genetic variants are associated with various stone-forming phenotypes. Given the high recurrence rates and the difficulty in treating brushite stones, we elected to investigate the genetics of this population.

Overall, in our study, brushite stone formers have a high rate (80%) of genetic variants, with 40% having multiple variants. Interestingly, 10 (67%) patients had variants associated with hypophosphatemic rickets, of which 60% harbored *SLC34A3* variants associated with autosomal recessive HHRH. 24-hour urine parameters demonstrated clinically meaningful differences in urine volume, calcium, and phosphate between those who harbored *SLC34A3* variants and those with other genetic variants associated with hypophosphatemic rickets. While a single monogenic predisposition would be convenient, it is likely that the genetic underpinnings of brushite stone disease is varied and complex. Comparison to those without any genetic variants could not be undertaken due to the limited number of patients without variants. This is the first study to directly assess for genetic variants amongst brushite stone formers.

Hereditary hypophosphatemic rickets with hypercalciuria, an autosomal recessive disease, has long been thought of as a rare disease. It is caused by homozygous or compound heterozygous (biallelic) pathogenic variants in the *SLC34A3* gene which encodes the sodium dependent phosphate transport protein 2c [16, 17]. Disease onset is classically in infancy or childhood and the phenotype is characterized by early onset rickets and short stature, with hypophosphatemia from proximal tubular wasting of phosphate leading to reduced serum PTH, and increased 1,25-Dihydroxyvitamin D (1,25(OH)2D) production in the kidney [18]. In turn, active Vitamin D, increases dietary calcium absorption leading to hypercalciuria while maintaining a normal serum calcium. Hyperphosphaturia with varying degrees of hypercalciuria can lead to brushite stone formation, nephrocalcinosis and sometimes progressive chronic kidney disease. Oral phosphorus supplementation is the treatment for hypophosphatemic rickets with hypercalciuria, as it normalizes serum phosphorus, reverses rachitic bone disease, and can variably lower 1,25(OH)2D levels and reduce hypercalciuria; though, it is not clear if unmonitored phosphate supplementation worsens nephrocalcinosis. Hypercalciuria and hyperphosphaturia, on the other hand, can be worsened by attempts to increase serum phosphorus with vitamin D analogs, increasing nephrocalcinosis and promoting chronic kidney disease [18, 19]. 

A number of studies have suggested that heterozygous carriers of pathogenic *SLC34A3* have higher rates of nephrolithiasis and nephrocalcinosis with an approximately 3-fold increased risk compared to the general population [17, 20]. Other observers have suggested that rare variants in *SLC34A3* confer an increased risk of kidney stone disease with monoallelic variants in *SLC4A3* falling into an “intermediate category of pathogenicity” that is insufficient to cause the fully penetrant recessive disease [21]. The prevalence of *SLC4A3* variants in our cohort also supports the suggestion that SLC34A3 variants increase the risk of stone disease.

Only a single participant in the present study was confirmed to be a pathogenic carrier. The associated gene, *CYP24A1*, encodes the enzyme 1,25-dihydroxyvitamin D(3) 24-hydroxylase which is involved with catabolism of 1,25(OH)2D [22]. Biallelic mutations result in autosomal recessive infantile hypercalcemia type 1 from lack of inactivation of 1,25 vitamin D. Hypercalcemia leads to hypercalciuria, polyuria and dehydration with resultant nephrocalcinosis, and kidney stone disease. Large genome wide association studies have uncovered various loci in stone formers including the *CYP24A1* associated locus (rs17216707) known to be involved with serum calcium levels and recurrent kidney stone disease [23], while smaller case series have also corroborated data to show variants in *CYP24A1* as a risk factor for stone formation [24]. 

Our study, along with others, help to explain the complicated interaction between genetic and molecular pathways underpinning kidney stone disease. Yet, caution should be taken when interpreting this data. Most variants demonstrate only a slight relative risk of stone formation ranging from 1.1 to 1.25 [25]. Polygenic risk scores that combine the individual effects of multiple single nucleotide polymorphisms (SNPs) have shown greater utility in predicting kidney stone disease with an odds ratio of 1.3 as compared to using a single significant SNP [26]. Continued research into the genetics behind kidney stone disease will help to improve such risk scoring systems and allow for personalized medical care.

Genetics are the presumed explanation for familial stone disease, yet family history itself is a complex topic which deserves further study, including parental modeling [27]. The learned behaviors from parents regarding dietary selection and eating behaviors (including the timing, frequency and volume of food consumed) should not be overlooked and may also contribute to familial-associated stone formation.

The American Urologic Association (AUA) and European Association of Urology (EAU) currently do not recommend any form of genetic testing or screen as part of the evaluation for stone formers, given the limited understanding of the contribution of genetics to stone disease, underscoring the need for more dedicated studies. With a broader knowledge, the first consideration will be who to recommend for testing. Recent studies have demonstrated genetic variations amongst children and young adults (≤ 25 years old) at approximately 12–21% [4, 5, 28]. Given the lifespan in a such a population and the morbidity of recurrent stone disease, even a marginal diagnostic rate would seem acceptable to recommend testing. Similarly, patients with specific disease factors regardless of age, such as brushite stone composition, could also be considered if the diagnostic rates were sufficiently high. Geraghty et al. suggests considering genetic testing in adults >25 years if there is a clinical suspicion of metabolic disorder and/or recurrence (≥ 2 episodes), bilateral disease, or strong family history [6]. Improved understanding of genetic variants and their stone-forming phenotypes may help tailor the type or aggressiveness of medical therapy for an individual patient. Ultimately, it will likely take a genotype-specific intervention or prognosis (e.g. renal failure) in this population to drive payment for such testing.

There are several limitations to our study. First, the small size of the study, non-randomized nature of patient selection, and lack of patient diversity limit the generalizability of the results. Validation in large cohorts is needed. Second, the identification of VUS amongst this population is correlative and does not necessarily imply causality. Third, as a large referral center, we often participate in the care of patients who have been cared for over many years at other facilities without stone analysis performed. Thus, we are unable to comment on whether the current cohort have always been brushite stone formers or have converted to brushite stone formation. Last, the presentation of urinary parameters for comparison is hypothesis generating at best and is further limited by the small number of subjects and non-standardized dietary consumption on the dates of collection. Though the analysis was performed as exploratory only, it does show high number of genetic variants, which may be evaluated through further study.

## Conclusions

Brushite stone formers harbor high numbers of genetic variants of unknown significance, many of which are associated with monogenic causes of nephrolithiasis. Specifically, variants in autosomal recessive hereditary hypophosphatemic rickets with hypercalciuria are particularly overrepresented in this cohort. Future studies should involve validation in larger cohorts as well as the assessment of dietary and medical interventions for specific genotypes.

## Supplementary Information

Below is the link to the electronic supplementary material.

Supplementary Material 1

## Data Availability

No datasets were generated or analysed during the current study.

## References

[1] Anderegg MA, Olinger EG, Bargagli M, Geraghty R, Taylor L, Nater A, Bruggmann R, Sayer JA, Vogt B, Schaller A, Fuster DG (2024) Prevalence and characteristics of genetic disease in adult kidney stone formers. Nephrol Dial Transplant 39(9):1426–1441. 10.1093/ndt/gfae074 38544324 10.1093/ndt/gfae074 PMC11483609

[2] Resnick M, Pridgen DB, Goodman HO (1968) Genetic predisposition to formation of calcium oxalate renal calculi. N Engl J Med 278(24):1313–8. 10.1056/NEJM196806132782403 5648597 10.1056/NEJM196806132782403

[3] Curhan GC, Willett WC, Rimm EB, Stampfer MJ (1997) Family history and risk of kidney stones. J Am Soc Nephrol 8(10):1568–73. 10.1681/ASN.V8101568 9335385 10.1681/ASN.V8101568

[4] Halbritter J, Baum M, Hynes AM, Rice SJ, Thwaites DT, Gucev ZS, Fisher B, Spaneas L, Porath JD, Braun DA, Wassner AJ, Nelson CP, Tasic V, Sayer JA, Hildebrandt F (2015) Fourteen Monogenic genes account for 15% of nephrolithiasis/nephrocalcinosis. J Am Soc Nephrol 26(3):543–551 Epub 2014 Oct 8. PMID: 25296721; PMCID: PMC4341487 25296721 10.1681/ASN.2014040388 PMC4341487

[5] Geraghty R, Lovegrove C, Howles S, Sayer JA (2024) Role of genetic testing in kidney stone disease: a narrative review. Curr Urol Rep 25(12):311–323. 10.1007/s11934-024-01225-5 39096463 10.1007/s11934-024-01225-5 PMC11374836

[6] Siener R, Herwig H, Rüdy J, Schaefer RM, Lossin P, Hesse A (2022) Urinary stone composition in Germany: results from 45,783 stone analyses. World J Urol 40(7):1813–1820. 10.1007/s00345-022-04060-w. (**Epub 2022 Jun 6. PMID: 35666268; PMCID: PMC9236976**) 35666268 10.1007/s00345-022-04060-w PMC9236976

[7] Daudon M, Jungers P, Bazin D, Williams JC Jr. (2018) Recurrence rates of urinary calculi according to stone composition and morphology. Urolithiasis 46(5):459–470. 10.1007/s00240-018-1043-0Epub 2018 Feb 1. PMID: 29392338; PMCID: PMC6711148 29392338 10.1007/s00240-018-1043-0 PMC6711148

[8] Klee LW, Brito CG, Lingeman JE (1991) The clinical implications of brushite calculi. J Urol 145(4):715–8. 10.1016/s0022-5347(17)38432-x 2005685 10.1016/s0022-5347(17)38432-x

[9] Krambeck AE, Handa SE, Evan AP, Lingeman JE (2010) Profile of the brushite stone former. J Urol 184(4):1367–1371. 10.1016/j.juro.2010.05.094 20719342 10.1016/j.juro.2010.05.094 PMC3173741

[10] Siener R, Pitzer MS, Speller J, Hesse A (2023) Risk profile of patients with brushite stone disease and the impact of diet. Nutrients 15(18):4092. 10.3390/nu15184092PMID: 37764875; PMCID: PMC10534559 37764875 10.3390/nu15184092 PMC10534559

[11] Mansilla MA, Sompallae RR, Nishimura CJ, Kwitek AE, Kimble MJ, Freese ME, Campbell CA, Smith RJ, Thomas CP (2021) Targeted broad-based genetic testing by next-generation sequencing informs diagnosis and facilitates management in patients with kidney diseases. Nephrol Dial Transplant 36(2):295–305. 10.1093/ndt/gfz173 31738409 10.1093/ndt/gfz173 PMC7834596

[12] Richards S, Aziz N, Bale S, Bick D, Das S, Gastier-Foster J, Grody WW, Hegde M, Lyon E, Spector E, Voelkerding K, Rehm HL, ACMG Laboratory Quality Assurance Committee (2015) Standards and guidelines for the interpretation of sequence variants: a joint consensus recommendation of the American College of Medical Genetics and Genomics and the Association for Molecular Pathology. Genet Med 17(5):405–424. 10.1038/gim.2015.30 25741868 10.1038/gim.2015.30 PMC4544753

[13] Tavtigian SV, Harrison SM, Boucher KM, Biesecker LG (2020) Fitting a naturally scaled point system to the ACMG/AMP variant classification guidelines. Hum Mutat 41(10):1734–1737. 10.1002/humu.24088 32720330 10.1002/humu.24088 PMC8011844

[14] Palsson R, Indridason OS, Edvardsson VO, Oddsson A (2019) Genetics of common complex kidney stone disease: insights from genome-wide association studies. Urolithiasis 47(1):11–21. 10.1007/s00240-018-1094-2Epub 2018 Dec 6. PMID: 30523390 30523390 10.1007/s00240-018-1094-2

[15] Cao X, Jiang M, Guan Y, Li S, Duan C, Gong Y, Kong Y, Shao Z, Wu H, Yao X, Li B, Wang M, Xu H, Hao X (2025) Trans-ancestry GWAS identifies 59 loci and improves risk prediction and fine-mapping for kidney stone disease. Nat Commun 16(1):3473. 10.1038/s41467-025-58782-7PMID: 40216741; PMCID: PMC11992175 40216741 10.1038/s41467-025-58782-7 PMC11992175

[16] Chi Y, Zhao Z, He X, Sun Y, Jiang Y, Li M, Wang O, Xing X, Sun AY, Zhou X, Meng X, Xia W (2014) A compound heterozygous mutation in *SLC34A3* causes hereditary hypophosphatemic rickets with hypercalciuria in a Chinese patient. Bone 59:114–121 (**Epub 2013 Nov 16. PMID: 24246249**) 24246249 10.1016/j.bone.2013.11.008

[17] Dasgupta D, Wee MJ, Reyes M, Li Y, Simm PJ, Sharma A, Schlingmann KP, Janner M, Biggin A, Lazier J, Gessner M, Chrysis D, Tuchman S, Baluarte HJ, Levine MA, Tiosano D, Insogna K, Hanley DA, Carpenter TO, Ichikawa S, Hoppe B, Konrad M, Sävendahl L, Munns CF, Lee H, Jüppner H, Bergwitz C (2014) Mutations in SLC34A3/NPT2c are associated with kidney stones and nephrocalcinosis. J Am Soc Nephrol 25(10):2366–2375. 10.1681/ASN.2013101085 24700880 10.1681/ASN.2013101085 PMC4178443

[18] Bergwitz C, Miyamoto KI (2019) Hereditary hypophosphatemic rickets with hypercalciuria: pathophysiology, clinical presentation, diagnosis and therapy. Pflugers Arch 471(1):149–163. 10.1007/s00424-018-2184-2 30109410 10.1007/s00424-018-2184-2

[19] Zhu Z, Bo-Ran Ho B, Chen A, Amrhein J, Apetrei A, Carpenter TO, Lazaretti-Castro M, Colazo JM, McCrystal Dahir K, Geßner M, Gurevich E, Heier CA, Simmons JH, Hunley TE, Hoppe B, Jacobsen C, Kouri A, Ma N, Majumdar S, Molin A, Nokoff N, Ott SM, Peña HG, Santos F, Tebben P, Topor LS, Deng Y, Bergwitz C (2024) An update on clinical presentation and responses to therapy of patients with hereditary hypophosphatemic rickets with hypercalciuria (HHRH). Kidney Int. ;105(5):1058–1076. doi: 10.1016/j.kint.2024.01.031. Epub 2024 Feb 15. Erratum in: Kidney Int. 2024;106(1):159. 10.1016/j.kint.2024.05.005. PMID: 38364990; PMCID: PMC11106756 10.1016/j.kint.2024.01.031 PMC11106756 38364990

[20] Brunkhorst M, Brunkhorst L, Martens H, Papizh S, Besouw M, Grasemann C, Turan S, Sikora P, Chromek M, Cornelissen E, Fila M, Lilien M, Allgrove J, Neuhaus TJ, Eltan M, Espinosa L, Schnabel D, Gokce I, González-Rodríguez JD, Khandelwal P, Keijzer-Veen MG, Lechner F, Szczepańska M, Zaniew M, Bacchetta J, Emma F, Haffner D (2025) Presentation and outcome in carriers of pathogenic variants in *SLC34A1* and *SLC34A3* encoding sodium-phosphate transporter NPT 2a and 2c. Kidney Int 107(1):116–129. 10.1016/j.kint.2024.08.035 39461557 10.1016/j.kint.2024.08.035

[21] Sadeghi-Alavijeh O, Chan MMY, Moochhala SH, Genomics England Research Consortium, Howles S, Gale DP, Böckenhauer D (2023) Rare variants in the sodium-dependent phosphate transporter gene *SLC34A3* explain missing heritability of urinary stone disease. Kidney Int 104(5):975–984. 10.1016/j.kint.2023.06.019 37414395 10.1016/j.kint.2023.06.019

[22] Schlingmann KP, Kaufmann M, Weber S, Irwin A, Goos C, John U, Misselwitz J, Klaus G, Kuwertz-Bröking E, Fehrenbach H, Wingen AM, Güran T, Hoenderop JG, Bindels RJ, Prosser DE, Jones G, Konrad M (2011) Mutations in CYP24A1 and idiopathic infantile hypercalcemia. N Engl J Med 365(5):410–421. 10.1056/NEJMoa1103864 21675912 10.1056/NEJMoa1103864

[23] Howles SA, Wiberg A, Goldsworthy M, Bayliss AL, Gluck AK, Ng M, Grout E, Tanikawa C, Kamatani Y, Terao C, Takahashi A, Kubo M, Matsuda K, Thakker RV, Turney BW, Furniss D (2019) Genetic variants of calcium and vitamin D metabolism in kidney stone disease. Nat Commun 10(1):5175. 10.1038/s41467-019-13145-x 31729369 10.1038/s41467-019-13145-x PMC6858460

[24] Nesterova G, Malicdan MC, Yasuda K, Sakaki T, Vilboux T, Ciccone C, Horst R, Huang Y, Golas G, Introne W, Huizing M, Adams D, Boerkoel CF, Collins MT, Gahl WA (2013) 1,25-(OH)2D-24 hydroxylase (CYP24A1) deficiency as a cause of nephrolithiasis. Clin J Am Soc Nephrol 8(4):649–657 Epub 2013 Jan 4. PMID: 23293122; PMCID: PMC3613951 23293122 10.2215/CJN.05360512 PMC3613951

[25] Singh P, Harris PC, Sas DJ, Lieske JC (2022) The genetics of kidney stone disease and nephrocalcinosis. Nat Rev Nephrol 18(4):224–240. 10.1038/s41581-021-00513-4Epub 2021 Dec 14. PMID: 34907378 34907378 10.1038/s41581-021-00513-4

[26] Paranjpe I, Tsao N, Judy R, Paranjpe M, Chaudhary K, Klarin D, Forrest I, O’Hagan R, Kapoor A, Pfail J, Jaladanki S, Chaudhry F, Vaid A, Duy PQ, Charles Bronfman Institute of Personalized Medicine Genomics Team; Regeneron Genomics Team, He JC, Glicksberg BS, Coca SG, Gupta M, Do R, Damrauer SM, Nadkarni GN (2020) Derivation and validation of genome-wide polygenic score for urinary tract stone diagnosis. Kidney Int 98(5):1323–1330 Epub 2020 Jun 12. PMID: 32540406; PMCID: PMC7606592 32540406 10.1016/j.kint.2020.04.055 PMC7606592

[27] Mahmood L, Flores-Barrantes P, Moreno LA, Manios Y, Gonzalez-Gil EM (2021) The influence of parental dietary behaviors and practices on children’s eating habits. Nutrients 13(4):1138. 10.3390/nu13041138PMID: 33808337; PMCID: PMC8067332 33808337 10.3390/nu13041138 PMC8067332

[28] Gefen AM, Sethna CB, Cil O, Perwad F, Schoettler M, Michael M, Angelo JR, Safdar A, Amlie-Wolf L, Hunley TE, Ellison JS, Feig D, Zaritsky J (2023) Genetic testing in children with nephrolithiasis and nephrocalcinosis. Pediatr Nephrol 38(8):2615–2622. 10.1007/s00467-023-05879-0Epub 2023 Jan 23. PMID: 36688940; PMCID: PMC11071637 36688940 10.1007/s00467-023-05879-0 PMC11071637

