# Supplementary material for: Variants of unknown significance are common in brushite stone formers undergoing genetic testing for nephrolithiasis
Source: Urolithiasis. 2025 Sep 29;53(1):183. doi: 10.1007/s00240-025-01859-1 (PMC12479612; doi:10.1007/s00240-025-01859-1)
Supplement: Supplementary file 1 — Supplementary Material 1 [file 240_2025_1859_MOESM1_ESM.docx]

Supplemental Table 1. List of genes tested in the KidneySeq^TM^ platform (IIHG, Iowa City, IA) nephrolithiasis/nephrocalcinosis subpanel

| **KidneySeq™ Nephrolithiasis/Nephrocalcinosis** | | |
| --- | --- | --- |
| **Disease** | **Inheritance Pattern** | **Gene(s)** |
| APRT deficiency (stones and ESRD) | AR | *APRT* |
| Bartter syndrome | AD | *CaSR* |
|  | AR | *KCNJ1, SLC12A1* |
| Cystinuria | AD, AR | *SLC3A1, SLC7A9* |
| Dent disease | XLR | *CLCN5, OCRL* |
| Familial hypocalciuric hypercalcemia | AD | *CaSR* |
| Fanconi syndrome, generalized proximal defect | AD | *EHHADH, HNF4A* |
|  | AR | *ATP7B, CTNS, FAH, SLC34A1* |
|  | XLR | *CLCN5* |
| Hypercalcemia, infantile | AR | *CYP24A1* |
| Hypercalciuria | AD | *ADCY10* |
| Hyperoxaluria, primary | AR | *AGXT, GRHPR, HOGA1* |
| Hypocalcemia, autosomal dominant | AD | *CASR* |
| Hypomagnesemia with hypercalciuria | AR | *CLDN16, CLDN19* |
| Hypophosphatasia, adult* | AD, AR | *ALPL* |
| Hypophosphatasia, child* | AR | *ALPL* |
| Hypophosphatasia, infantile* | AR | *ALPL* |
| Hypophosphatemic rickets | AD | *FGF23, SGK3* |
|  | AR | *DMP1, ENPP1, SLC34A3, VDR* |
|  | XLR | *CLCN5, PHEX* |
| Hypouricemia, renal | AD | *SLC2A9* |
|  | AR | *SLC22A12* |
| Lesch-Nyhan syndrome | XLR | *HPRT1* |
| Nephrolithiasis/osteoporosis, hypophosphatemic | AD | *SLC9A3R1* |
| Renal tubular acidosis, distal | AD, AR | *ATP6V0A4, ATP6V1B1, ATP6V1C2, FOXI1, SLC4A1, WDR72* |
| Renal tubular acidosis, proximal | AD | *EHHADH, HNF4A* |
|  | AR | *ATP7B, CTNS, FAH, SLC34A1, SLC4A4* |
|  | XLR | *ATP7B, CLCN5* |
| Xanthine oxidase deficiency | AR | *XDH* |
| AD=autosomal dominant, AR= autosomal recessive, XLR= X-linked recessive | | |
